# Charlson comorbidity index applied to shunted idiopathic normal pressure hydrocephalus

**DOI:** 10.1038/s41598-023-32088-4

**Published:** 2023-03-29

**Authors:** Petra M. Klinge, Kevin L. Ma, Owen P. Leary, Rahul A. Sastry, Shanzeh Sayied, Ollin Venegas, Thomas Brinker, Ziya L. Gokaslan

**Affiliations:** 1 Department of Neurosurgery, Rhode Island Hospital, Warren Alpert Medical School of Brown University, 593 Eddy St, APC 6, Providence, RI 02903 USA; 2 Department of Surgery, Perelman School of Medicine, University of Pennsylvania, Philadelphia, PA 19104 USA; 3 Department of Anesthesiology and Critical Care Medicine, University of New Mexico School of Medicine, Albuquerque, NM 87131 USA; 4 Department of Neurosurgery, Medical School Hannover, Hannover, Germany

**Keywords:** Neurology, Neurological disorders

## Abstract

A series of epidemiological studies have shown the limited life expectancy of patients suffering from idiopathic normal pressure hydrocephalus (iNPH). In most cases, comorbid medical conditions are the cause of death, rather than iNPH. Though it has also been shown that shunting improves both life quality and lifetime. We sought to investigate the utility of the Charlson comorbidity index (CCI) for improved preoperative risk–benefit assessment of shunt surgery in individual iNPH cases. 208 shunted iNPH cases were prospectively investigated. Two in-person follow up visits at 3 and 12 months assessed postoperative clinical status. The correlation of the age adjusted CCI with survival was investigated over the median observation time of 2.37 years (IQR 1.16–4.15). Kaplan Meier statistics revealed that patients with a CCI score of 0–5 have a 5-year survival rate of 87%, compared to only 55% in patients with CCI > 5. Cox multivariate statistics revealed that the CCI was an independent predictor of survival, while common preoperative iNPH scores (modified Rankin Scale (mRS), gait score, and continence score) are not. As expected, mRS, gait, and continence scores improved during the postoperative follow up period, though relative improvement on any of these was not predicted by baseline CCI. The CCI is an easily applicable preoperative predictor of survival time in shunted iNPH patients. The lack of a correlation between the CCI and functional outcome means that even patients with multiple comorbidities and limited remaining lifetime may appreciate benefit from shunt surgery.

## Introduction

Idiopathic normal pressure hydrocephalus (iNPH) is a disease of the elderly, and it is well established that iNPH patients can clinically benefit from shunt surgery^1,2^. While recent epidemiological and clinical studies have shown the limited life expectancy of iNPH patients^3–6^, it has also been reported that shunting may improve the life expectancy^4^, and furthermore this beneficial effect may be diminished in iNPH patients who are not treated by shunting in a timely manner^7^.

Comorbid medical conditions have been recognized as the cause of death in most fatal iNPH cases^5,8^. A large epidemiological registry study revealed that iNPH itself was coded as underlying cause of death in less than 5% of iNPH patients^4^. In the preoperative setting, comorbidities can be quantitatively assessed in individual patients by applying the Charlson comorbidity index (CCI)^9^, and the age-adjusted CCI allows for the long-term survival prediction^10^. Accordingly, the CCI comprises a useful tool for weighing the potential benefits versus harms of a medical or surgical treatment during the remaining lifespan^11^. While the CCI has been validated to provide such useful information on the remaining lifetime in numerous diseases, it has not yet been validated for this purpose in iNPH^12^.

At this point, because shunting for iNPH must be considered as elective surgery, neurosurgeons often face a difficult decision making for shunting in individual patients with a variety of comorbidities. Currently, it is not possible to reliably predict the survival or functional outcome of individual patients in light of preoperative comorbidity burden^13^. Taking a quantitative approach to this issue, we applied the age-adjusted CCI in a cohort of 208 shunted iNPH patients. Our results demonstrate that the CCI was significantly predictive of survival time in this population. Though it was not predictive of the functional outcome of shunt surgery for iNPH.

## Methods

A single-surgeon consecutive cohort of 208 iNPH cases who were shunted from 2015 to 2021 was prospectively studied. All patients fulfilled the diagnostic criteria of iNPH as diagnosed by clinical examination and MRI^14^. A subset of patients with equivocal radiographic or clinical findings underwent an additional high-volume lumbar tap test, though a negative tap test was not considered a strict exclusion from shunting^15^.

Commonly used and validated scores evaluated based on clinical examination were used to document the preoperative symptom severity, including the modified Rankin score (mRS), iNPH gait score, and iNPH continence score (Table 1)^1,2,13,14^. Those tests were also applied at the 3- and 12-months follow-up visits. In addition, based on clinician and patient reported data, clinically significant impairment of cognition was documented in a binary fashion at the preoperative visit and its improvement at the two follow up visits. Comorbidity burden was assessed at the preoperative visit applying the 19-item CCI version^9,12^, which was age adjusted (Table 2)^10^.

**Table 1:** Clinical Scores.

Score	Continence	Gait	mRS
0			No symptoms
1	Normal	Normal	No disability despite symptoms
2	Urgency without incontinence	Slight disturbance of tandem walk and turning	Slight disability- able to look after own affairs without assistance
3	Infrequent incontinence	Wide based gait with sway, without foot corrections	Moderate disability requiring some help but able to walk without assistance
4	Frequent incontinence	Tendency to fall, with foot correction	Moderately severe disability unable to walk, attend to body needs without assistance
5	Bladder incontinence	Walking with cane	Severe disability, bedridden, incontinent, and requiring constant nursing care
6	Bladder and bowl incontinence	Bi-manual support is needed	
7		Aided- by another person	
8		Wheelchair bound

**Table 2:** Charlson age adjusted comorbidity index.

Variable	Score	Prevalence, n (%)
Age		
51–60	1	6 (3%)
61–70	2	42(20%)
71–80	3	106(51%
81–90	4	54 (26%)
91+	5	0
Myocardial infarction	1	26 (12%)
Congestive heart failure	1	12 (5.8%)
Peripheral vascular disease	1	32 (15%)
Cerebrovascular disease	1	30 (14%)
Dementia	1	184 (90%)
Chronic pulmonary disease	1	27 (13%)
Rheumatological disease	1	9 (4.3%)
Peptic ulcer disease	1	9 (4.3%)
Liver disease		
Mild	1	1 (0.5%)
Moderate or severe	3	2 (1.0%)
Diabetes		
Uncomplicated	1	34 (16%)
Chronic complications	2	22 (11%)
Hemi- Quadriplegia	2	2 (1.0%)
Chronic renal disease	2	15 (7.2%)
Leukemia/Lymphoma	2	4 (1.9%)
Cancer		
Any tumor	2	67 (32%)
Metastatic solid tumor	6	2 (1.0%)
AIDS	6	0 (0%)

Patient survival time was assessed in October 2022 using a combination of the hospital’s electronic medical records and web-based obituary searches. All cases in whom death or survival could not be confirmed were censored at the date of the last clinical visit. A total of 41 deaths were confirmed.

This study was IRB approved (Lifespan Institutional Review Board, Providence RI 201,819 USA) and patients’ informed consent was waived (Ref. #1,345,067). All methods were performed in accordance with the relevant guidelines and regulations.

Availability of data and materials: Raw data are available upon reasonable request to the corresponding author.

### Statistics

For survival time analysis, univariate and multivariate cox proportional hazards regression, and Kaplan Meier analysis with log rank testing, were performed. For evaluation of the clinical follow-up the Friedman rank sum test with post-hoc pairwise Wilcoxon rank sum test with Bonferroni correction were applied. Generalized linear modeling (GLM) was applied to assess whether the CCI is predictive for clinical outcome. All statistical analyses were completed using R software v4.2.1.

## Results

### Study population

The median age was 75 years (IQR 71–80). 208 iNPH cases were treated either by a ventriculoperitoneal (n = 205) or by a ventriculopleural shunt (n = 3). 98 cases (47%) were female.

The median observation time for the survival statistics was 2.37 years (IQR 1.16–4.15). 93% of cases (n = 194) at the 3-month follow-up, and 73% (n = 152) at the 12-month follow-up, were seen in person in our outpatient clinic. Considering that at the 12-month visit already 12 patients were deceased, the overall 12-month follow-up rate was 79%. The clinical scoring scales are shown in Table 1.

The age adjusted CCI scale and the prevalence of underlying pathologies of the study group are shown in Table 2.

At the preoperative visit the median (IQR) CCI was 6 (5–8), gait score was 4 (4–6), continence score was 3 (2–4) and mRS was 2 (2–3). Cognitive impairment was reported in 184 of 208 patients (88%).

### Survival time and clinical scoring

**Figure 1:**
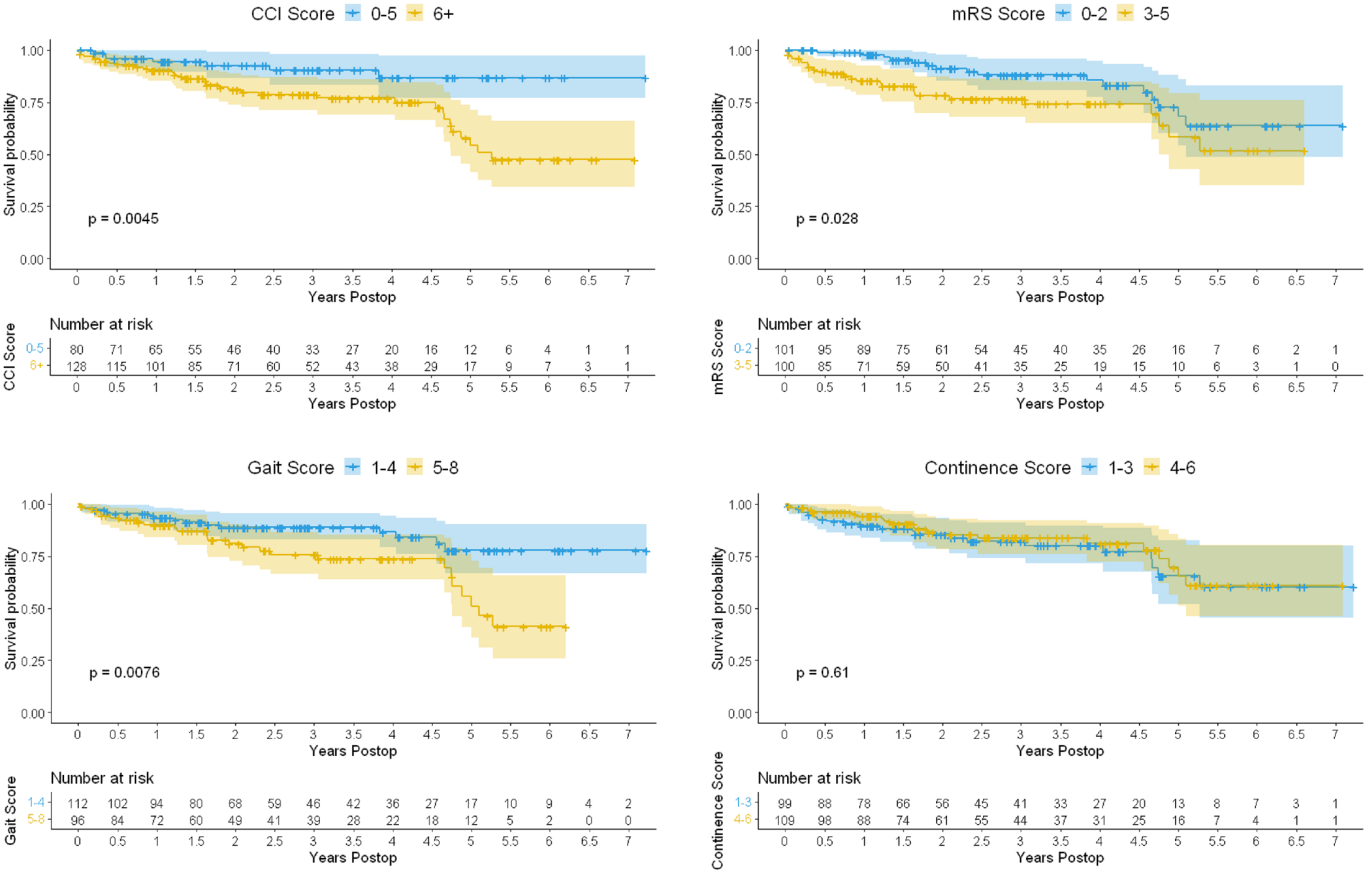
Kaplan–Meier analysis of survival time versus CCI, mRS, gait and continence score. The log rank test revealed significant differences between the low and high score groups of CCI, mRS and gait, but not continence score. The 95% confidence intervals and censoring of cases are shown.

Kaplan Meier survival analysis was performed applying the preoperative score findings including age-adjusted CCI, mRS, gait and continence scores (Fig. 1).

For the Kaplan–Meier curves, preoperative results were used and the ordinal symptom scales were dichotomized for better visualization as has been reported by others: mRS score 0–2 versus 3–5 , gait score 1–4 versus 5–8, and continence score 1–3 versus 4–6^4^. The CCI was dichotomized at a score of 5. That score was chosen considering the median age of the cases (70–80 years = 3 CCI points), and the high prevalence of cognitive impairment which was coded as dementia (1 CCI point). Assuming another minor comorbidity (1 CCI point), a total score of CCI ≤ 5 is characteristic of relatively healthy patients, compared to patients characterized by higher preoperative comorbidity burden (CCI > 5). Kaplan Meier statistics revealed that patients with a CCI score of 0–5 have a 5-year survival rate of 87%, compared to only 55% in patients with CCI > 5.

Applying univariate cox regression and the full ordinal scores, the age-adjusted CCI, the gait and the mRS scores reached a statistical significance (*p *< 0.05). Of these, only the CCI remained significantly correlated with survival time on multivariate Cox regression analysis (Table 3).

**Table 3:** Survival analysis applying the full scales.

Univariate cox regression				Multivariate cox regression		
	HR	95%CI	P	HR	95%CI	*p*
CCI	1.269	1.11–1.452	0.000508	1.243	1.087–1.422	0.00149
mRS	1.622	1.086–2.424	0.0183	1.515	0.887–2.607	0.13335 n.s
Gait score	1.346	1.077–1.681	0.00888	1.062	0.7859–1.422	0.69599 n.s
Continence score	1.061	0.89–1.351	0.59 n.s	1.018	0.8229–1.260	0.86625 n.s

### Functional outcome

**Figure 2:**
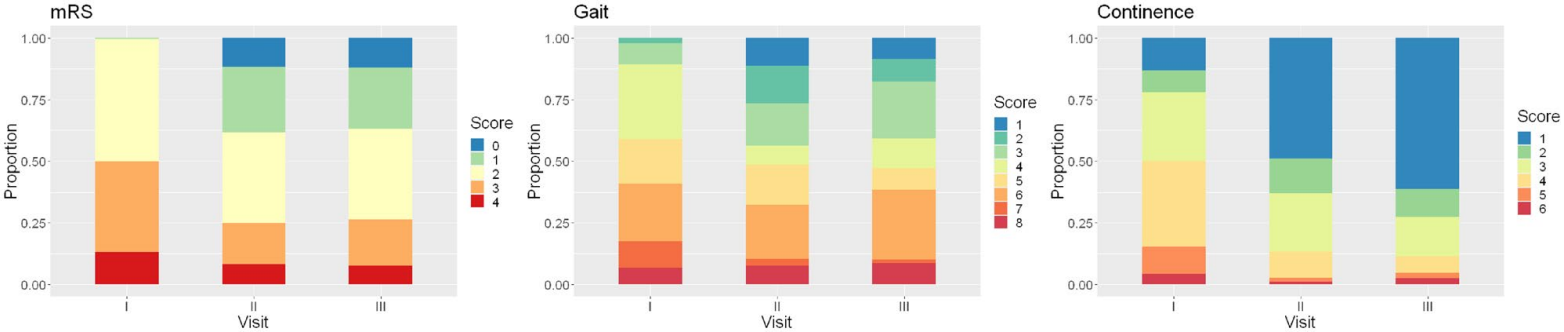
Stacked proportional bar graphs of mRS, gait and continence scores. Postoperative follow-up examinations show a significant improvement of the three categories. Yellow indicates moderate impairment, red and orange are worse, blue, and green are better.

mRS, gait and continence scores improved significantly from preoperative scoring to both the 3-month and 12-month follow-up visits. Figure 2 demonstrates that the improvement of clinical symptoms already appreciable at 3 months following surgery became even more pronounced at the 12-months follow-up.

Statistical analysis applying the Friedman test revealed significant postoperative improvements in terms of all three clinical scores. The paired post-hoc Wilcoxon test revealed the improvements in all scores between the preoperative and 3-month visits, and between the preoperative and 12-month visits, to be statistically significant (Table 4). Improvement between 3-month and 12-month visits was significant for gait score (*p *= 0.049), but not for mRS or continence score.

**Table 4:** Clinical outcome.

Statistics	Friedman test	Paired post-hoc Wilcoxon test		
		Pre-Op to 3 M	Pre-Op to 12 M	3 M to 12 M
mRS	*P *= 9.099e−16	*P *= 2.0e−12	*P *= 1.9e−10	*P *= 0.78 n.s
Gait	*P *< 2.2e−16	*P *< 2.2e−16	*P *= 14.5e−16	*P *= 0.049
Continence	*P *< 2.2–16	*P *< 2.2e−16	*P *< 2.2e−16	*P *= 0.41 n.s

Compared to 88% of patients with cognitive impairment preoperatively, only 81 of 194 patients (42%) remained impaired at 3-month follow-up, and only 64 of 152 (42%) cases at the 12 month follow-up.

### Is there a relation between the improvement of clinical outcome scores 3 and 12 months after surgery and the preoperative CCI?

Applying generalized linear modeling (GLM), age-adjusted CCI was statistically correlated with improvement of the mRS score at the 3-month visit, though the statistical analysis indicates higher CCI scores with improvement of the mRS and the regression coefficient is quite low (R^2^ = 0.06). At the 12-month visit, CCI was not related to improvement of any of the clinical scores (Table 5).

**Table 5:** Multivariate linear regression.

	Estimate	Std.error	t-value	Pr( >|t|)
3 Months FU				
Intercept	6.44356	0.29764	21.649	< 2e−16***
MRS	− 0.67549	0.19755	− 3.419	0.000806 ***
Gait score	0.16381	0.11015	1.487	0.139049 n.s
Continence score	− 0.03516	0.09821	0.358	0.720794 n.s
12 Months FU				
Intercept	6.42312	0.32105	20.007	< 2e−16 ***
MRS	0.10247	0.22686	− 0.452	0.652 n.s
Gait score	0.01088	0.11059	− 0.098	0.922 n.s
Continence score	− 0.14866	0.11239	− 1.323	0.188 n.s

**Figure 3:**
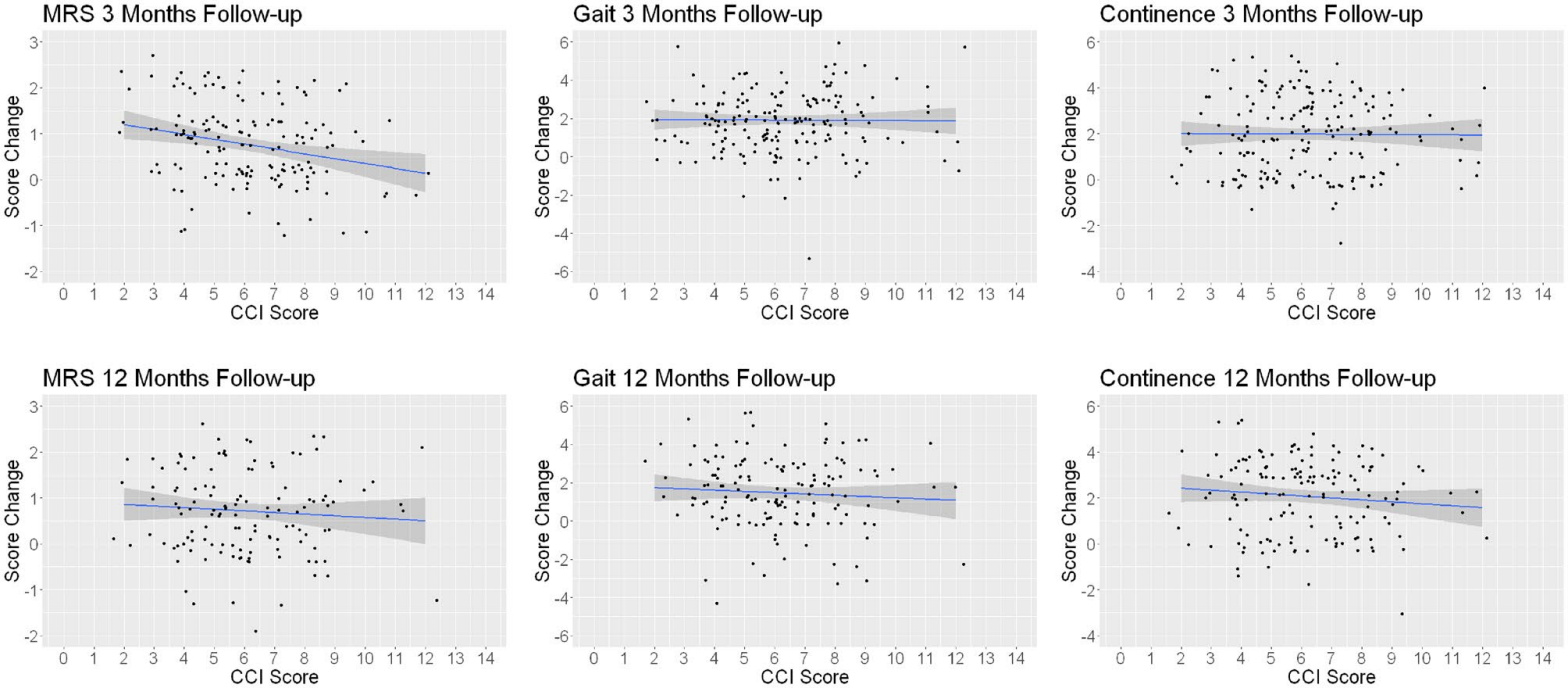
Linear regression analysis of changes of the clinical score at the follow-up as compared with the preoperative scores was not related to the preoperative CCI. The regression line is shown and in addition the individual data visualized as jitter plot. The jitter plot is like a scatter plot but adds random noise to better see the information contained in the data, when there is a lot of overplotting.

Applying linear regression analysis for correlations between the CCI and the improvements of each individual clinical score, we did not find any meaningful correlation (Table 6, Fig. 3).

**Table 6:** Univariate linear regression.

	Univariate linear regression			
	3 months		12 months	
	R2	P	R2	*P*
MRS	0.06	0.05	0.006	0.38
Gait	7.1e−5	0.91	0.006	0.35
Continence	5.4e−5	0.92	0.01	0.22

## Discussion

This is the first study revealing that the CCI provides a quantitative estimate of the remaining lifetime in individual shunted iNPH cases. Further, and consistent with the most recent literature^16^, we confirm that CCI is not a predictor of functional outcome across multiple accepted outcome scales in this population.

### Life expectancy and comorbidity in iNPH

It is a rather novel understanding that the life expectancy in iNPH is quite limited. Early reports were based on small clinical case series^6,17^ but comprehensive studies addressing this issue have been conducted just more recently^4,8,18^. It has been shown that in untreated cases with radiologically proven ventricular enlargement, the hazard ratio (HR) for death was significantly increased (HR = 3.8, as compared with the general population)^3^. In shunted iNPH, the hazard ratio is improved to 3.3^6^. Also, it has been shown that delayed shunting of diagnosed iNPH further reduces life expectancy^7^. Cumulatively, these data support the notion that mortality is increased in iNPH as compared with the general population. Our survival data are consistent with these previously published data.

Importantly, while acknowledging the mortality risk in iNPH patients, previous studies have also recognized that iNPH is not a deadly disease by itself. Among 979 treated INPH cases from the Swedish Hydrocephalus Quality Registry, iNPH was coded as cause of death in only 5% of the fatal cases, implicating comorbidity burden instead as a frequent cause. Observations from other studies have also supported this notion^5,6,8,18^. While some other studies have tried to correlate specific comorbidities, especially cerebrovascular disorders, to the onset of iNPH, the etiological relationship between iNPH and other underlying disease processes remains inconclusive. Preexisting factors like obesity, diabetes, hypertension, and others appear to have some an impact^4,5,19^. However, it appears that at this point it is premature to draw any conclusions and we believe that further research is needed in these areas.

### Charlson comorbidity index

First introduced in 1987, the CCI includes of a total of 19 disease conditions weighted according to relative impact on mortality^9^. Since its inception, CCI has been used in numerous studies investigating the remaining life expectancy following treatment of a variety of diseases^12^. The index was age-adjusted in 1994 by adding 1 score point for each decade above an age of 50 years. It was shown that the inclusion of age is important in time series studies which extend the observation time to more than a few months^10^. Therefore, we used the age-adjusted CCI score for our study of iNPH patients over a follow-up period of years. Until today several modifications of the original score were published; for example the score has been adjusted to ICD and other coding requirements^12^.

The Charlson index is a unique measure of comorbidity burden, since it can be easily computed by means of a questionnaire, including by patients themselves^12^. Our results indicate that the Charlson comorbidity index can categorize individual iNPH patients in terms of life expectancy to a statistically significant extent. We demonstrate that an age-adjusted CCI score of > 5 identifies iNPH patients with a much more limited life expectancy than those with a score between ≤ 5. Our data indicate that iNPH cases with a maximum CCI score of 5 survive one year with a probability of 95%, and 5 years with a probability of 87%. Those numbers drop in cases with a CCI score of 6 and higher to 90% and 54% respectively.

Importantly, multivariate Cox analysis revealed that, while the CCI is an independent predictor of survival in shunted iNPH patients, comorbidity burden does not predict functional improvement as assessed by mRS, gait and continence scores.

### Functional Outcome following shunting for iNPH

Neurological outcome in the study cohort was significantly improved comparing the preoperative visit with the 3 and 12 months follow up visits. The improvement was statistically significant with each of the functional outcome scores (mRS, gait, continence). This is in line with existing literature, including overall lack of further improvement from 3-month to 12-month outcome. Importantly, applying generalized linear modeling, we found no correlation between the CCI (dependent variable) and the degree of improvement in mRS, gait and continence scores (independent variables). These findings confirmed previous observations of others^16^. However, that lack of a correlation between CCI and functional improvement means that a patient with a CCI score higher than 5 may very well benefit from shunting for the remaining lifetime, information that may be helpful to providers and patients alike when weighing the risks and potential benefits of shunting.

### Limitations

Data of this prospective study were collected at the preoperative and the two follow up visits. Our population’s mRS scores ranged from 1 to 4, with no scores of 5 (bedridden) at the preoperative or follow up visits. This likely reflects referral trends, such that mRS = 5 cases were not referred to our outpatient clinic because they were not considered as candidate for invasive treatment procedures. Our overall loss to follow-up rate is similar to other iNPH studies, though the validity of our results is affected especially at the second follow-up visit^20^. As compared to the Swedish epidemiological studies which have access to the “national cause of death registry”, we relied on documented death records in electronic medical records of the hospital and online obituaries. Because all patients with unknown survival status were censored at the last documented clinical visit (visits at other departments of our hospital and affiliated hospitals were included), our survival analysis did probably miss a limited number of deceased cases. However, the survival rate could be calculated within reasonable 95% confidence intervals up to seven years follow up. Finally, there exists the issue that the CCI may overestimate life expectancy because it was assessed only once prior to shunt surgery, not accounting for potential increases in patient comorbidity burden during the follow up period. This issue is currently under investigation^11^, and meanwhile cannot be addressed with routine statistical procedures.

Eventually, those limitations indicate the need for future studies which should address specific issues, including: a more detailed examination of neurodegenerative comorbidities not included within the CCI but which may influence survival and further analysis of how comorbidities influence mRS and the gait scale. While not presented in the current analysis, future analyses might also employ improved collection of morbidity data to enable a more precise calculation of survival time with respect to each incremental point-wise increase in CCI. Finally, as practice potentially shifts towards alternative CSF diversion procedures, such as lumboperitoneal shunting, the application of the CCI scale in iNPH cases treated by these alternative procedures merits further study.

## Conclusion

The CCI is an easily applicable predictor of survival time in shunted iNPH patients. The lack of a correlation between the CCI and functional outcome means that even patients with multiple comorbidities and limited remaining lifetime may very well benefit from shunt surgery.

## References

[1] Klinge P, Hellstrom P, Tans J, Wikkelso C (2012). European i NPHMSG: One-year outcome in the European multicentre study on iNPH. Acta Neurol. Scand..

[2] Klinge P, Marmarou A, Bergsneider M, Relkin N, Black PM (2005). Outcome of shunting in idiopathic normal-pressure hydrocephalus and the value of outcome assessment in shunted patients. Neurosurgery.

[3] Jaraj D, Wikkelso C, Rabiei K, Marlow T, Jensen C, Ostling S, Skoog I (2017). Mortality and risk of dementia in normal-pressure hydrocephalus: A population study. Alzheimers Dement.

[4] Andren K, Wikkelso C, Sundstrom N, Israelsson H, Agerskov S, Laurell K, Hellstrom P, Tullberg M (2020). Survival in treated idiopathic normal pressure hydrocephalus. J. Neurol..

[5] Israelsson H, Larsson J, Eklund A, Malm J (2020). Risk factors, comorbidities, quality of life, and complications after surgery in idiopathic normal pressure hydrocephalus: Review of the INPH-CRasH study. Neurosurg. Focus.

[6] Malm J, Kristensen B, Stegmayr B, Fagerlund M, Koskinen LO (2000). Three-year survival and functional outcome of patients with idiopathic adult hydrocephalus syndrome. Neurology.

[7] Andren K, Wikkelso C, Hellstrom P, Tullberg M, Jaraj D (2021). Early shunt surgery improves survival in idiopathic normal pressure hydrocephalus. Eur. J. Neurol..

[8] Pyykko OT, Nerg O, Niskasaari HM, Niskasaari T, Koivisto AM, Hiltunen M, Pihlajamaki J, Rauramaa T, Kojoukhova M, Alafuzoff I (2018). Incidence, comorbidities, and mortality in idiopathic normal pressure hydrocephalus. World Neurosurg..

[9] Charlson ME, Pompei P, Ales KL, MacKenzie CR (1987). A new method of classifying prognostic comorbidity in longitudinal studies: Development and validation. J. Chronic. Dis..

[10] Charlson M, Szatrowski TP, Peterson J, Gold J (1994). Validation of a combined comorbidity index. J. Clin. Epidemiol..

[11] Van Hemelrijck M, Ventimiglia E, Robinson D, Gedeborg R, Holmberg L, Stattin P, Garmo H (2022). Population-based estimates of age and comorbidity specific life expectancy: A first application in Swedish males. BMC Med. Inform. Decis. Mak..

[12] Charlson ME, Carrozzino D, Guidi J, Patierno C (2022). Charlson comorbidity index: A critical review of clinimetric properties. Psychother. Psychosom..

[13] Sundstrom N, Rydja J, Virhammar J, Kollen L, Lundin F, Tullberg M (2022). The timed up and go test in idiopathic normal pressure hydrocephalus: A nationwide study of 1300 patients. Fluids Barriers CNS.

[14] Hellstrom P, Klinge P, Tans J, Wikkelso C (2012). A new scale for assessment of severity and outcome in iNPH. Acta Neurol Scand.

[15] Wikkelso C, Hellstrom P, Klinge PM, Tans JT (2013). European i NPHMSG: The european iNPH multicentre study on the predictive values of resistance to CSF outflow and the CSF tap test in patients with idiopathic normal pressure hydrocephalus. J. Neurol. Neurosurg. Psychiatry.

[16] Valsecchi N, Mantovani P, Piserchia VA, Giannini G, Cevoli S, Aspide R, Oppi F, Milletti D, Cortelli P, Elder BD (2022). The role of simultaneous medical conditions in idiopathic normal pressure hydrocephalus. World Neurosurg..

[17] Raftopoulos C, Massager N, Baleriaux D, Deleval J, Clarysse S, Brotchi J (1996). Prospective analysis by computed tomography and long-term outcome of 23 adult patients with chronic idiopathic hydrocephalus. Neurosurgery.

[18] Junkkari A, Sintonen H, Danner N, Jyrkkanen HK, Rauramaa T, Luikku AJ, Koivisto AM, Roine RP, Viinamaki H, Soininen H (2021). 5-Year health-related quality of life outcome in patients with idiopathic normal pressure hydrocephalus. J. Neurol..

[19] Israelsson H, Carlberg B, Wikkelso C, Laurell K, Kahlon B, Leijon G, Eklund A, Malm J (2017). Vascular risk factors in INPH: A prospective case-control study (the INPH-CRasH study). Neurology.

[20] Dettori JR (2011). Loss to follow-up. Evid. Based Spine Care J..

